# Effectiveness of hochuekkito for colorectal cancer management: protocol for the multicenter randomized controlled AMATERAS study

**DOI:** 10.1007/s00384-026-05197-5

**Published:** 2026-07-28

**Authors:** Yoshinaga Okugawa, Takahito Kitajima, Tadanobu Shimura, Hiroki Imaoka, Mikio Kawamura, Akira Yamamoto, Aya Kawamoto, Kosuke Mizukoshi, Hiroyuki Fujikawa, Takashi Iwata, Susumu Saigusa, Eiki Ojima, Yasuhiro Inoue, Yasuhiko Mohri, Yuji Toiyama

**Affiliations:** 1 Department of Genomic Medicine, Mie University Hospital, Tsu City, Mie, Japan; 2 Department of Gastrointestinal and Pediatric Surgery, Division of Reparative Medicine, Institute of Life Sciences, Mie University Graduate School of Medicine, Tsu City, Mie, Japan; 3 Department of Gastrointestinal and General Surgery, Mie Prefectural General Medical Center, Yokkaichi City, Mie, Japan; 4 Department of Surgery, Kuwana City Medical Center, Kuwana City, Mie, Japan; 5 Department of Surgery, IGA City General Hospital, Iga City, Mie, Japan; 6 Oncology Center, Aichi Medical University School of Medicine and Hospital, Nagakute City, Aichi, Japan; 7 Department of Surgery, Doshinkai Tohyama Hospital, Tsu City, Mie, Japan

**Keywords:** Hochuekkito, TJ-41, Systemic inflammation, Colorectal cancer

## Abstract

**Background:**

Systemic inflammation, a hallmark of cancer, is recognized as a key mechanism underlying malnutrition, sarcopenia, and cancer cachexia. Hochuekkito (TJ-41) is a traditional Japanese Kampo formula composed of ten medicinal plants, and several studies have suggested its anti-inflammatory effects in various diseases. Our recent retrospective study, supported by in vitro experiments, was the first to demonstrate an anti-inflammatory effect of TJ-41 in patients with unresectable or metastatic gastrointestinal cancer, including colorectal cancer (CRC). However, no prospective randomized multicenter clinical trial has evaluated TJ-41 in the palliative setting of malignancies, including CRC.

**Methods:**

Patients with unresectable or metastatic CRC who have received at least two prior systemic regimens or are intolerant to standard therapy, and who show an elevated C-reactive protein (CRP) level > 0.5 mg/dL on two consecutive occasions within two months before enrollment, will be eligible. After providing written informed consent, patients will be randomly assigned to the treatment group (standard treatment plus TJ-41) or control group (standard treatment alone). Patients in the treatment group will receive oral TJ-41 (Tsumura & Co., Tokyo, Japan) at 7.5 g/day, divided into three doses (2.5 g per dose), before, or between meals for 12 weeks. The primary endpoint is the difference in anti-inflammatory effect between groups, assessed by the change in CRP from baseline to 12 weeks. Secondary endpoints include the effects of TJ-41 on other inflammatory and nutritional parameters; patient-reported symptoms and quality of life (QOL) using QOL-ACD and EORTC QLQ-C30; cytokine-based anti-inflammatory and anticachectic effects using serial blood specimens; interactions with other appetite-stimulating agents or nutritional products; and effects on antitumor efficacy and adverse events (AEs). Each group will include 44 patients, with recruitment planned from July 1, 2024, to December 31, 2026.

**Discussion:**

This trial will evaluate whether TJ-41 can attenuate systemic inflammation related to host–tumor interactions in patients with unresectable or metastatic CRC.

**Trial registration:**

This study was registered in the Japan Registry of Clinical Trials (jRCT) on June 26, 2024 (jRCTs041240051).

## Background

Colorectal cancer (CRC) is the third most commonly diagnosed cancer and the second leading cause of cancer death worldwide [1]. Furthermore, more than half of CRC cases are diagnosed at an advanced stage. By this time, tumor cell dissemination has already occurred, despite recent advances in screening protocols [2]. Although treatment options have improved substantially, including the development of novel chemotherapeutic and molecular agents, as well as technical advances in invasive treatment for metastatic lesions, recurrence and metastasis remain leading causes of death in CRC patients [1]. In addition, effective delivery of novel anticancer drug therapies for unresectable or metastatic CRC requires supportive care to maintain the patient’s general condition. Malnutrition and cancer cachexia, in particular, are associated with increased treatment-related toxicities and reduced ability to continue such therapies [3]. Based on these standpoints, there is an urgent need to develop a different treatment approach to improve clinical outcomes in these patients.

Systemic inflammation via host–tumor interaction, one of the hallmarks of cancer [4], has long been recognized as a trigger of cancer cachexia and is also gradually recognized as the cause of impaired body composition, including skeletal muscle loss, and malnutrition [5–8]. Our group has also previously revealed that elevated C-reactive protein (CRP) levels were an independent risk factor for the presence of preoperative myopenia in 308 CRC patients [9]. In fact, international consensus on the diagnostic criteria for malnutrition, which was established by the Global Leadership Initiative on Malnutrition (GLIM) consortium, explicitly states that a systemic inflammation is one etiological factor in malnutrition diagnosis [10]. Additionally, the Asian Wasting and Cachexia Diagnosis Criteria (AWGC) list elevated CRP as a diagnostic criterion and specify an optimal cutoff value greater than 0.5 [11]. Furthermore, our previous clinical study also clarified that mitigating CRP elevation through fish oilenriched immunonutrition contributes to better chemotherapy tolerance and survival outcomes, especially in unresectable or metastatic gastrointestinal cancer patients presenting with elevated CRP prior to intervention [12]. In light of these backgrounds, it is essential to consider developing supportive therapies that suppress systemic inflammation caused by cancer–host interactions. Such therapies could address malnutrition, sarcopenia, and cachexia, thereby improving continuity of cancer treatment, enhancing quality of life (QOL), and improving prognosis.

We previously reported that immunonutrition containing fish oil had anti-inflammatory effects in patients with unresectable or metastatic gastrointestinal cancer, including CRC [12, 13], who were undergoing systemic chemotherapy. This led to improvements in skeletal muscle mass, treatment continuity, and survival, particularly in patients with elevated CRP levels. However, adherence was poor; 51 out of 88 patients discontinued intake within the intervention period during six months [12]. Thus, we have continued to pursue the development of novel supportive therapies.

Hochuekkito (TJ-41) is a traditional Japanese Kampo medicine made from ten medicinal plants. It is commonly prescribed in Japan for anorexia and general fatigue. Clinical trials show that TJ-41 improves quality of life and immunological status in older adults [14–16], and in patients with chronic obstructive pulmonary disease, it mitigates systemic inflammation, prevents weight loss, and enhances nutritional status [17]. Additionally, several studies have revealed the anti-inflammatory effects of TJ-41 through in vitro and in vivo experiments [18–21]. We also recently assessed clinical data from 99 patients including CRC who received TJ-41 treatment for more than 3 months and in vivo experiments [22]. This study demonstrated that treatment with TJ-41 induced a significant, time-dependent decline in serum CRP levels, with the effect being particularly evident in patients with metastatic malignancies. During TJ-41 therapy, muscle mass was significantly better preserved in the subgroup with decreased CRP levels than in those with increased levels. Consistent with these clinical observations, in vivo experiments using a Colon-26 syngeneic mouse model showed that plasma levels of CRP, serum amyloid A, and interleukin-6 were significantly reduced in mice receiving TJ-41 compared with levels in controls. However, because this study is retrospective and lacks a control group, prospective interventional studies are needed to clarify the anti-inflammatory effects of TJ-41 and its associated outcomes in relation to malnutrition, sarcopenia, and cachexia.

This prospective interventional study aims to investigate whether the administration of TJ-41 contributes to an anti-inflammatory effect and maintenance of nutritional status in a palliative setting for patients with unresectable or metastatic CRC and systemic inflammation.

### Methods

**Fig. 1:**
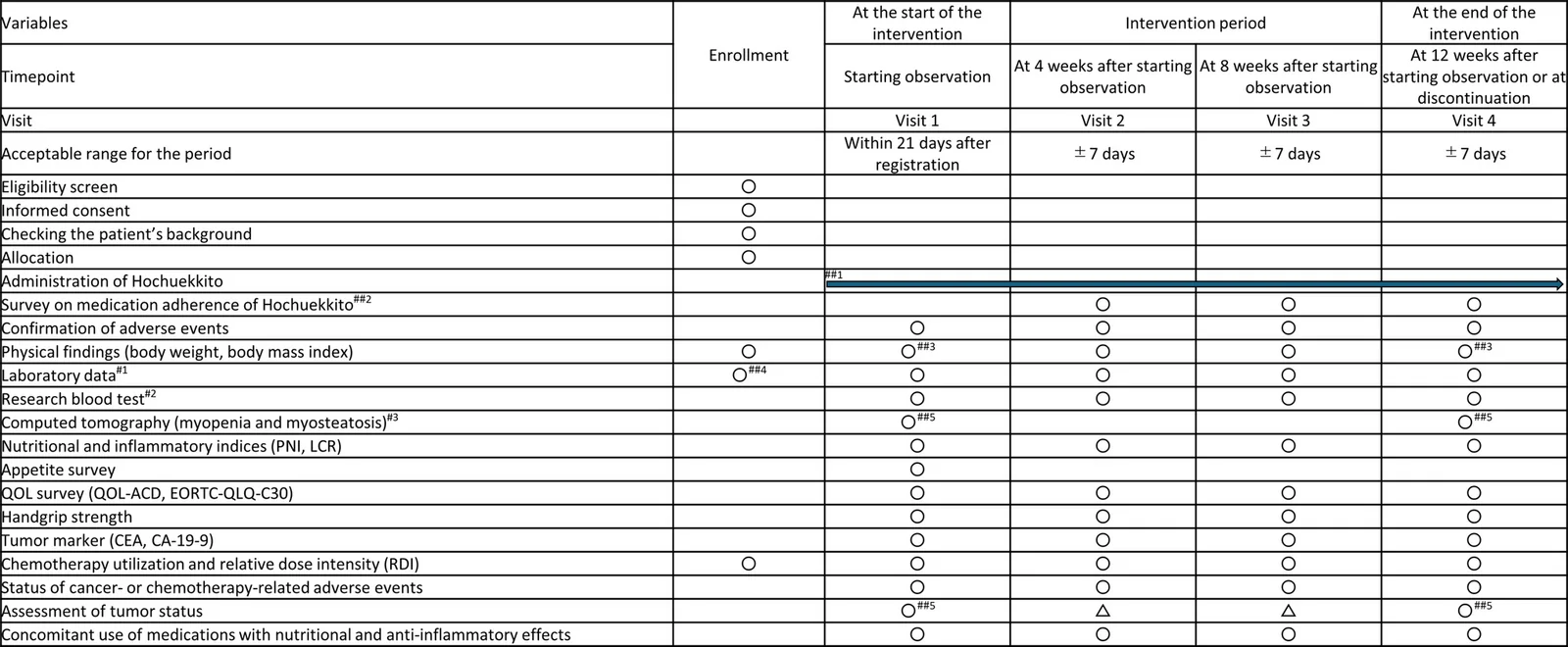
The schedule of enrolment, interventions, and assessments in the AMATERAS study. #1 RBC, WBC, Hb, Ht, PLT, TP, Alb, TLC, T-bil, AST, ALT, ALP, γ-GTP, K, Cr, and CRP. #2 SAA, IL-6, transthyretin, transferrin, RBP, lactate, pyruvate, and GDF-15. #3:CT-based assessments for myopenia (psoas mass index) and myosteatosis (intramuscular adipose tissue content and modified intramuscular adipose tissue content). ##1 Do not take the medication of the treatment start date; begin taking the medication the following morning. ##2 Only the Hochuekkito administration group will be investigated. ##3 At the start of treatment and 12 weeks after treatment initiation (or at discontinuation), confirm changes in body weight. ##4 CRP must be confirmed to be greater than 0.5 mg/dL on two consecutive occasions during routine clinical examinations within the past two months prior to registration. ##5 Imaging test results obtained within two weeks before or after the baseline date (the start of treatment, 12 weeks after the start of treatment, or discontinuation) are acceptable as substitutes. However, imaging tests should be performed on the day of the relevant visit whenever possible. △ Disease assessment may be added as part of routine care during cancer treatment if deemed necessary by the attending physician

**Fig. 2:**
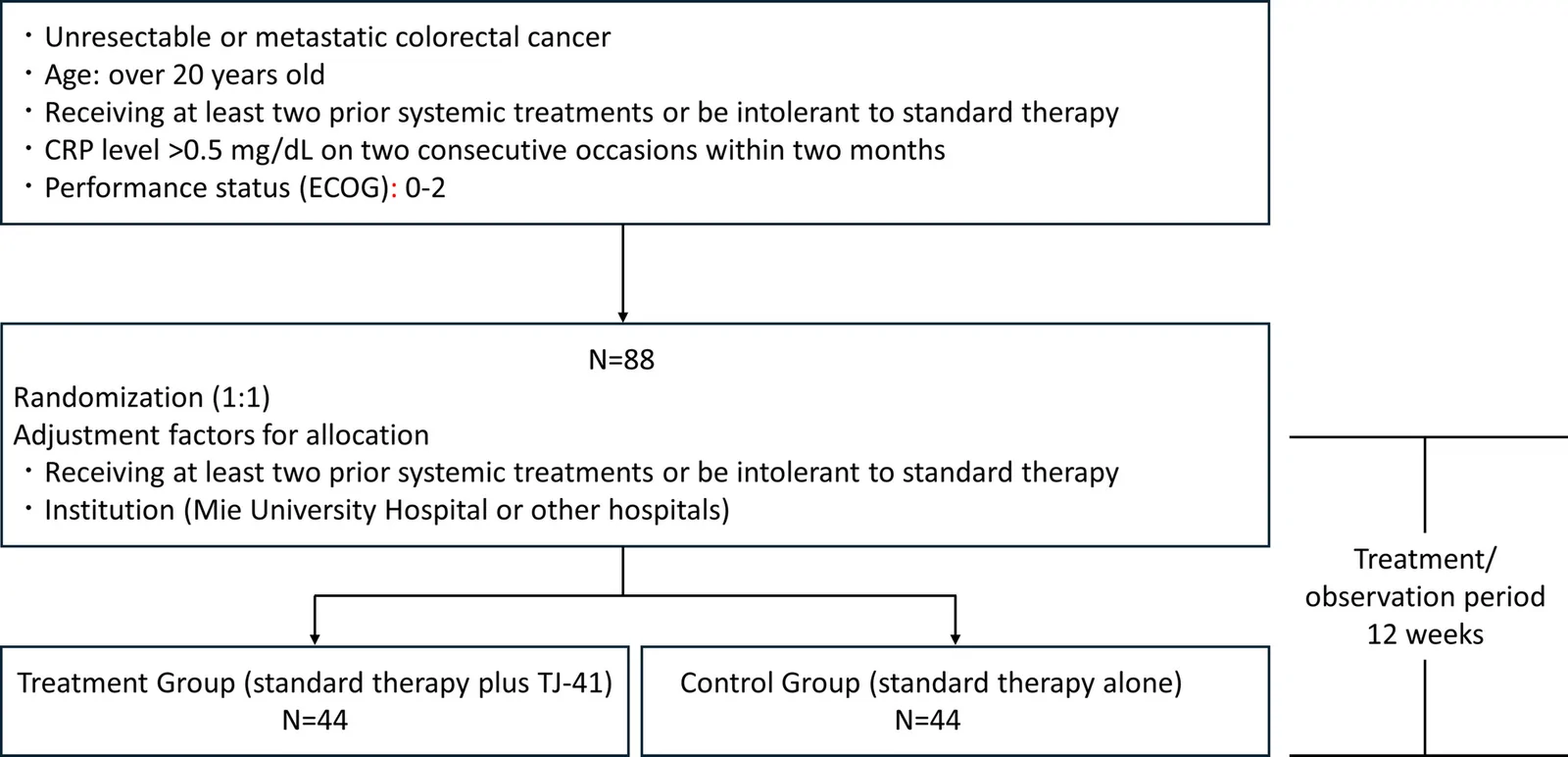
Flow diagram of the AMATERAS study. ECOG: Eastern Cooperative Oncology Group

The authors adhered to the SPIRIT recommendations when drafting this protocol (Fig. 1) [23]. The protocol is version 1.2, dated April 30, 2024. The CONSORT flowchart is shown in Fig. 2.

#### Trial design

This open-label, multicenter, randomized trial with a 1:1 allocation ratio is designed to evaluate TJ-41’s efficacy and safety for treating the anti-inflammatory effects of unresectable or metastatic CRC cancer (Fig. 2). This study is registered with the Japan Registry of Clinical Trials (jRCT) (jRCTs041240051). Ethical approval was obtained from the Mie University Hospital ethics committee and subsequently from the institutional review boards of all participating centers. Patients will be recruited from six hospitals across Japan, as listed in Table 1. The enrollment period is planned to last 2 years and 6 months, followed by a 3-month follow-up period. The maximum follow-up duration is 2 years and 9 months. This trial will be conducted in accordance with the ordinance of the Ministry of Health, Labour, and Welfare in Japan, Declaration of Helsinki, Ethical Guidelines for Medical and Biological Research Involving Human Subjects, Clinical Trials Act, and the guideline “Statistical Principles for Clinical Trials” [24]. The trial was also designed based on the guideline “Structure and Content of Clinical Study Reports” [25].

**Table 1:** The six participating institutes for AMATERAS study

Number	Center	Department	Investigator
1*	Mie University Hospital	Department of gastrointestinal and pediatric surgery	Yuji Toiyama
2	Mie Prefectural General Medical Center	Department of gastrointestinal and general surgery	Yasuhiko Mohri
3	Kuwana City Medical Center	Department of surgery	Kosuke Mizukoshi
4	IGA City General Hospital	Department of surgery	Susumu Saigusa
5	Aichi Medical University School of Medicine and Hospital	Oncology center	Takashi Iwata
6	Doshinkai Tohyama Hospital	Department of surgery	Yasuhiro Inoue

*Sponsor investigator

#### Trial eligibility

Patients with unresectable or metastatic CRC will be screened for eligibility. The inclusion criteria are as follows: Patients must be at least 20 years old at the time of consent. Patients who provided written consent to participate in this study. Patients must have received at least two prior systemic treatment regimens as adjuvant therapy and/or for metastatic disease (including at least one regimen for which disease progression was documented in the metastatic setting) or be intolerant to standard therapy. To mitigate the potential confounding influence of concurrent anticancer treatments on CRP levels, eligibility was restricted to patients with at least two prior regimens. Superior response rates in the first-line setting often lead to rapid tumor reduction and subsequent decreases in inflammation; therefore, such treatments could confound our results independently. Therefore, we enrolled a population that had undergone at least two prior regimens. This design was supported by our earlier retrospective observation of the anti-inflammatory activity of TJ-41 in later-line therapeutic settings [22]. Patients with a confirmed CRP level greater than 0.5 mg/dL on two consecutive occasions within two months prior to enrollment based on routine clinical laboratory testing. Patients must have an Eastern Cooperative Oncology Group (ECOG) performance status of 0 to 2 and be expected to be followed for at least 12 weeks after study enrollment (Fig. 2).

The exclusion criteria are as follows: patients with MSI-high or TMB-high colorectal cancer for whom immune checkpoint inhibitors are or have been used as standard therapy; patients with comorbidities that may affect nutritional or inflammatory status, such as chronic kidney disease or an autoimmune disease, are also excluded; patients with infectious diseases requiring antibiotic therapy; patients with a stoma who have diarrhea interfering with activities of daily living; patients who have undergone surgical procedures within the past four weeks, or who are scheduled to undergo surgery during the study period; patients with a history of gastrectomy; patients who have taken Kampo medicines within two weeks prior to the scheduled date of visit 1; patients for whom oral food intake is difficult owing to gastrointestinal disorders, such as intestinal obstruction or dysphagia; patients who have difficulty taking Kampo medicine; patients with moderate or severe hepatic impairment (Child–Pugh class B or C) are also excluded; patients whose routine clinical laboratory test results from the two months prior to enrollment meet any of the following criteria: AST, ALT, or γ-GTP greater than five times the institutional upper limit of normal (ULN), total bilirubin or creatinine greater than three times the institutional ULN, potassium < 3.0 mmol/L or > 5.5 mmol/L; patients with pleural effusion or ascites; patients with synchronous or metachronous double (multiple) cancers; patients with serious comorbid conditions (e.g., cardiac, hematologic, or metabolic diseases); patients with a history of severe drug hypersensitivity or allergy, particularly to TJ-41; pregnant or breastfeeding women; women who may be pregnant; and patients who wish to become pregnant during the study period and patients who, at the discretion of the principal investigator or coinvestigator, are deemed otherwise ineligible to participate in this study.

Patients at the six participating hospitals will be screened. Those who meet all the eligibility criteria will be asked to participate in the study. After receiving sufficient information about the study, patients will decide whether to participate. Written informed consent will then be obtained from all those who agree to enroll.

#### Registration

The central registration office at WDB COCO Co., Ltd. will receive an eligibility report form. (Tokyo, Japan). Patients who meet all the inclusion criteria and none of the exclusion criteria will be randomized in a 1:1 ratio to either the treatment group (standard therapy plus TJ-41) or control group (standard therapy alone). Randomization will be performed using a minimization method with stratification by treatment course at the time of enrollment (receiving at least two prior systemic treatment regimens as adjuvant therapy and/or for metastatic disease or be intolerant to standard therapy), and the institution (Mie University Hospital or other hospitals) as variables before intervention. Although baseline CRP levels were initially considered as a stratification factor, the number of variables was limited to ensure statistical robustness in relation to the target sample size. Consequently, treatment history and institution were prioritized as the most clinically influential prognostic factors. To account for potential differences in baseline inflammation, baseline CRP levels will be included as a covariate in the primary statistical analysis. Investigators will not be informed of the details of the randomization program. After randomization, the registration center at WDB COCO will provide each investigator with information on the required follow-up assessments. This trial will be conducted at academic or general hospitals in Japan, and the list of participating sites will be available from the AMATERAS study office at Mie University Hospital.

#### Interventions

Protocol treatment must be initiated within 21 days of case registration. If initiation cannot occur within 21 days, informed consent must be reobtained, eligibility must be reconfirmed, and specified tests must be performed. Patients in the treatment group will receive TJ-41 (Tsumura & Co., Tokyo, Japan) orally at 7.5 g per day, divided into 3 doses (2.5 g/dose per packet), before or between meals for 12 weeks of standard treatment. TJ-41 is a powdered hot water-extract consisting of the following 10 components: astragalus root (Astragali radix), 4.0 g; *Atractylodes lancea* rhizome (*Atractylodis lanceae* rhizoma), 4.0 g; ginseng (ginseng radix), 4.0 g; Japanese Angelica root (*Angelicae* radix), 3.0 g; Bupleurum root (*Bupleuri* radix), 2.0 g; jujube (*Zizyphi fructus*), 2.0 g; citrus unshiu peel (*Aurantii nobilis pericarpium*), 2.0 g; Glycyrrhiza (*Glycyrrhizae* radix), 1.5 g; Cimicifuga rhizome (*Cimicifugae* rhizoma), 1.0 g; and ginger (Zingiberis rhizoma), 0.5 g, as previously described [22]. TJ-41 is manufactured by Tsumura & Co. If a dose is missed, the patient may take it as soon as they remember. However, if the next scheduled dose is imminent, skip the missed dose and take the next dose at the regular time. Participant adherence will be monitored by having participants return unused medication and through questionnaires based on their self-reported medication status at each visit. Patients in the control group will receive standard cancer treatment only, without TJ-41, for 12 weeks.

Regarding concomitant therapy and supportive care, prohibited concomitant medications include Kampo preparations other than the investigational product, immune checkpoint inhibitors, and antibacterial agents. There are no restricted concomitant medications, and no concomitant therapies are either prohibited or restricted beyond those specified above.

There are no specific provisions defined regarding follow-up treatment after this study is completed. The principal investigator or coinvestigator will discuss subsequent treatment options with each study participant and determine the appropriate course of care.

#### Primary and secondary endpoints

The primary endpoint is the comparison of the anti-inflammatory effect between the TJ-41 administration group (treatment group) and the nonadministration, as assessed by the change in CRP levels from baseline to 12 weeks after initiation of treatment. The rationale for selecting the change in CRP at 12 weeks as primary endpoint for this study is based on the results from prior research [1]. In this study, we retrospectively assessed the serial changes in CRP levels over a 12-week period in patients taking Hochuekkito (TJ-41) and confirmed that, after 12 weeks, a significant decrease of CRP level was observed, particularly in cancer patients with distant metastases. The CRP change rate at 12 weeks is defined as the CRP value at 12 weeks divided by the CRP value at treatment initiation. The date of treatment initiation is used as the baseline for this calculation (change rate = CRP at 12 weeks after treatment initiation/CRP at treatment initiation).

The secondary outcome measures the following: the effects of TJ-41 on other inflammatory and nutritional parameters; the effects of TJ-41 on patient-reported symptoms and QOL; the anti-inflammatory and anticachectic effects of TJ-41 at the cytokine level; the combined effects of TJ-41 and other appetite-stimulating agents or nutritional products; and the effects of TJ-41 on antitumor efficacy and the occurrence of adverse events (AEs). Changes in inflammatory and nutritional parameters before and after TJ-41 administration were assessed using results from routine clinical examinations. Blood tests (RBC, WBC, Hb, Ht, PLT, TP, Alb, TLC, T-bil, AST, ALT, ALP, γ-GTP, K, Cr, and CRP); physical findings (body weight and BMI); nutritional and inflammatory indices (prognostic nutrition index and lymphocyte-CRP ratio); CT-based assessments (myopenia [psoas mass index] and myosteatosis [intramuscular adipose tissue content and modified intramuscular adipose tissue content]); and handgrip strength. Changes in symptoms and QOL were assessed using QOL questionnaires such as QOL-ACD and EORTC QLQ-C30 before and after treatment in the TJ-41 administration group versus the non-administration group. Changes in blood cytokine levels related to anti-inflammatory and anticachectic effects were assessed using research blood tests for serum amyloid A, interleukin-6, transthyretin, transferrin, retinol-binding protein, lactate, pyruvate, and growth differentiation factor-15 before and after treatment in the TJ-41 administration versus non-administration groups. Furthermore, participants may receive various systemic and supportive therapies during the intervention period, including agents that could affect inflammatory or nutritional status. To address these potential confounders, we will evaluate the use of concomitant medications, such as steroids and nonsteroidal anti-inflammatory drugs (NSAIDs), as well as anamorelin and medical nutritional products covered by Japanese national health insurance. Our aim is to clarify the comparative impact of these treatments on anti-inflammatory and nutritional outcomes in the TJ-41 and control groups. Additionally, to account for confounders related to anticancer therapy and disease progression, we will evaluate tumor status and adverse events before and after treatment. Specifically, we will compare parameters including tumor markers (CEA, CA19-9), anticancer agent use, relative dose intensity (RDI), chemotherapy-related adverse events, and tumor response via imaging analysis between the two groups. Adverse events related to cancer or chemotherapy will be assessed using the Common Terminology Criteria for Adverse Events (CTCAE) v5.0 [26]. Tumor response to standard concomitant treatment will be evaluated using RECIST guidelines (v1.1) [27].

#### Criteria for Discontinuation of Study Drug and Observation Termination

The principal investigator or co-investigator shall discontinue administration of the study drug if they determine that continued administration is impossible for any of the following reasons: 1) when the research subject requests discontinuation of the study drug administration, 2) when continuation of the study drug administration is deemed undesirable owing to worsening of the underlying disease, 3) when continuation of the study drug administration is difficult owing to worsening of a complication, 4) when continuation of study drug administration is difficult owing to disease or other reasons, and 5) when the principal investigator or subinvestigator deems it appropriate to discontinue study drug administration for other reasons. If discontinuation occurs owing to the onset of a disease or other condition, follow-up shall be conducted until the subject recovers to their original condition as much as possible.

In contrast, observation will be terminated in the following cases: (1) if the research subject requests to withdraw from the study or withdraws consent; (2) if, after enrollment, it is found that the subject does not meet the eligibility criteria; (3) if pregnancy is confirmed; (4) if the subject ceases to visit the study site because of relocation or other reasons; (5) if the entire study is discontinued; and (6) if the principal investigator or co-investigator considers it appropriate to discontinue the study for any other reason such as death due to disease progression.

The date, reason, and course of discontinuation shall be recorded in the medical record and the electronic data capture (EDC) system. Necessary tests shall be performed at the time of discontinuation or withdrawal to evaluate efficacy and safety.

#### Sample size

A prior study [22] reported that the standard deviation of the log-transformed CRP change rate was 1.5 three months after TJ-41 administration in patients with unresectable CRC receiving cancer drug therapy. Based on this result, the present study used the log-transformed CRP change rate at three months as the primary endpoint to determine the sample size. The CRP change rate is defined as follows: CRP change rate = CRP (3 months)/CRP (0 months). For the TJ-41 combination group (treatment group), the outcome measure is defined as log (CRP change rate) = log (CRP (3 months)/CRP (0 months)). In the standard treatment-only group (control group), the outcome measure is defined as follows, assuming an additional effect on the actual CRP value at three months: log ([CRP (3 months) + effect size]/CRP (0 months)). The effect size for the difference in CRP levels between the two groups at 3 months was assumed to be 0.3. This value was selected based on our prior research [22], which demonstrated an effect size of 0.21 for CRP reduction following 3 months of TJ-41 administration. Furthermore, an effect size of 0.3 is considered clinically meaningful, as supported by our previous study on fish-oil enriched immunonutrition in patients with stage IV gastrointestinal cancer; that study indicated that CRP levels increase significantly during disease progression in the nonintervention groups [12]. Accordingly, we adopted an effect size of 0.3 to determine the minimum required sample size while ensuring a conservative estimate of the treatment effect. Under this assumption, the corresponding difference in the mean log-transformed CRP change rate between the two groups was estimated to be approximately 1. Assuming equal variances between the two groups and a common standard deviation of 1.5, as well as a 1:1 allocation ratio, the required sample size to detect a mean difference of 1 between groups was calculated using an unpaired two-sample *t*-test with a two-sided significance level of 5% and 80% power. Under these assumptions, the required sample size was estimated to be 35 patients per group. Furthermore, accounting for a potential dropout rate of approximately 20% during the observation period, the total target sample size was increased accordingly. The final planned sample size was set at 88 patients, with 44 in the TJ-41 combination group (treatment group) and 44 in the standard treatment-only group (control group).

#### Safety

Adverse events (AEs) are defined as any undesirable event occurring to a subject during the study, regardless of whether they are related to the investigational intervention. AEs of all grades to CTCAE v5.0 [26], reported either spontaneously by the subject or observed by the investigator or study staff, will be systematically recorded.

Serious adverse events (SAEs) are defined as any of the following: death; a life-threatening event (at the time of occurrence); an event requiring hospitalization or prolongation of existing hospitalization; an event resulting in permanent or significant disability or incapacity; an event causing a congenital anomaly or birth defect in offspring; or any other medically important event or effect judged to be serious by the principal investigator or a sub-investigator. However, this definition excludes adverse events that are known symptoms, signs, or diseases attributable to the underlying cancer; known adverse drug reactions to the anticancer agents currently being administered and listed as expected adverse events in the latest package insert or Drug Information Form for the relevant anticancer drug; and abnormal laboratory values that fall within the expected range of side effects of the anticancer agents currently being administered and are listed in the latest package insert or Drug Information Form for the relevant anticancer drug. Such events shall be classified as non-serious adverse events. SAEs will be reported to the accredited Medical Ethics Review Committee via a website within 15 days. This includes SAEs resulting in death or life-threatening events, as well as all other SAEs suspected to be related to the conduct of clinical research. Reporting will continue for the duration of the study. All AEs will be monitored until they are resolved or stabilized. An independent Data Safety Monitoring Board will oversee safety data throughout the study.

#### Data management, monitoring and auditing

In accordance with Good Clinical Practice, the investigators and clinical research coordinators will maintain individual source documents for each participant. These documents will include informed consent forms, medical records, laboratory reports, and case report forms (CRFs). All adverse events will be documented in the CRFs and followed up on prospectively until they are resolved or stabilized. The investigators will oversee all aspects of trial data management. Instead of convening a formal independent data monitoring committee, an independent medical advisor has been appointed to periodically review safety, feasibility, and any emerging issues. All trial data and relevant source documents will be archived for at least five years after the study’s completion.

An Efficacy and Safety Evaluation Committee (ESEC), composed of clinicians, statisticians, and ethics experts, has been established to enhance the quality of this trial. The ESEC maintains independence from the research team and objectively evaluates efficacy and safety. Conducted annually, it leads to critical decisions: “continue the trial,” “continue the trial with protocol modifications,” or “discontinue the trial.” Monitoring is performed by the WDB COCO based on the CRF and EDC system.

#### Statistical analysis

Statistical analyses, including the creation of tables and figures for the study report, will be conducted by the study statistician under blinded conditions, when feasible. The primary and secondary endpoint analyses will primarily be based on the full analysis set (FAS). To confirm the robustness of the results, additional analyses will be performed using the per-protocol set (PPS). Safety endpoint analyses will be conducted using the safety analysis set (SAS). The FAS includes all enrolled participants except those who did not attend any post-baseline visits after the start of treatment. Therefore, participants who discontinued the study prior to week 12 were included in the FAS, provided they had at least one post-baseline visit. The PPS is defined as the subset of participants who meet all of the following criteria: (1) no major protocol deviations, (2) receipt of at least two-thirds of the prescribed total dose of TJ-41 in the treatment group, and (3) no use of prohibited concomitant medications. Where missing values occur in longitudinal data endpoints within the FAS, we will apply multiple imputation to address these gaps. To evaluate potential bias due to missing data and ensure the robustness of our findings, we will conduct sensitivity analyses, comparing the results derived from the imputed datasets with those from a complete-case analysis.

The SAS for adverse events excludes participants who did not visit the clinic after registration and for whom no analyzable safety data are available. For the analysis of TJ-41–related adverse reactions, the analysis population excludes the following: (1) participants assigned to the TJ-41 group who did not attend any visits after the start of treatment and for whom no analyzable data are available and (2) participants assigned to the TJ-41 group for whom it was subsequently confirmed that TJ-41 was never administered.

For the primary endpoint, the CRP change rate will be log-transformed. The mean and standard deviation will be calculated for each group, and a Student’s *t*-test will be applied.　Categorical variables will be analyzed using cross-tabulation and Fisher’s exact test. For safety evaluation items (e.g., incidence of diseases or adverse events), cross-tabulations by group will be prepared, and Fisher’s exact test will be performed. The significance level for all statistical tests will be set at 5% (two-sided). All statistical analyses will be performed using validated statistical software.

#### Protocol amendments

Any modifications to the study protocol made during the trial must be submitted to the Certified Review Board of Mie University Hospital for approval by the principal investigator. After approval is granted, the principal investigator will promptly notify the lead investigators at each participating site of the changes and their rationale. The lead investigators will then submit the approved amendments to their respective institutional authorities for final approval.

#### Dissemination

The findings of this study will be disseminated through publication in peer-reviewed journals and presentation at scientific conferences. This ensures that no information identifying individual participants is disclosed. Authorship will be determined according to the criteria established by the International Committee of Medical Journal Editors (ICMJE).

#### Trial status

This study was approved by the Mie University Hospital Clinical Research Review Board based on the Clinical Trials Act on January 22, 2024 (S2023-004) and was registered with the Japan Registry of Clinical Trials on June 26, 2024 (jRCTs041240051). Participant recruitment began on July 1, 2024, and is ongoing as of January 1, 2026.

#### Role of the funding source

The funding agency was provided with the protocol and manuscript prior to submission for review of factual accuracy and funding-related information only and had no authority over study design, data analysis, manuscript content, or the decision to submit for publication.

## Discussion

This AMATERAS study is a multicenter, randomized controlled clinical trial to clarify the anti-inflammatory effect of cancer-harboring patients with unresectable or metastatic CRC.

To date, no clinical trials have investigated orally administered agents, including Kampo medicines, that may alleviate systemic inflammation by modulating host–tumor interactions at the palliative setting of malignancies, including CRC. Furthermore, this study included several secondary endpoints based on additional clinical data, handgrip strength, quality-of-life questionnaires, and translational research using serial blood samples to elucidate the clinical and biological impact of TJ-41 administration in these patients. This study’s results will be published as a research article and will provide novel evidence of the beneficial effects of TJ-41 in patients with unresectable or metastatic CRC.

This randomized controlled trial (RCT) aims to elucidate the anti-inflammatory effects of the Kampo formula TJ-41 in patients with colorectal cancer (CRC) through the serial assessment of biomarkers, including C-reactive protein (CRP), growth differentiation factor 15 (GDF-15), and inflammatory cytokines. Systemic inflammation is a recognized hallmark of malignancy, resulting from host–tumor interactions [4] and driving tumor initiation, progression, and metastasis [28–30]. In CRC patients, elevated inflammatory markers—particularly cytokines, such as IL-1β and IL-6, and serum CRP—are consistently associated with poor clinical outcomes [31–41]. Furthermore, systemic inflammation induces muscle wasting via cytokine-mediated appetite suppression and hypermetabolism. Growth differentiation factor 15 (GDF-15) has recently emerged as a key mediator of sarcopenia and cachexia [42, 43], with clinical trials of anti-GDF-15 antibodies demonstrating symptom mitigation in cancer patients [44]. Our recent finding of a significant positive correlation between preoperative CRP and GDF-15 levels suggests that systemic inflammation drives malnutrition and cachexia through GDF-15 signaling [45]. Accordingly, this prospective study will serially monitor these biomarkers to determine whether TJ-41 administration effectively modulates this inflammatory-cachectic axis in CRC patients.

Hochuekkito (TJ-41), a traditional Japanese Kampo medicine composed of ten herbs, possesses documented anti-inflammatory properties. Previous studies have shown that TJ-41 suppresses TNF-α production and inhibits inflammatory responses in the intestinal mucosa [18] and reduces rhinovirus-induced secretion of IL-6, TNF-α, IL-1, and IL-8 in tracheal epithelial cells [19]. In a COPD model, TJ-41 decreased inflammatory cell infiltration and attenuated lung histopathology [20]. Regarding malignancies, TJ-41 has been reported to significantly reduce circulating IL-6 levels and peritumoral macrophage IL-6 expression [21]. Our own clinical and in vivo data further corroborate these anti-inflammatory effects in cancer hosts [22]. Beyond its anti-inflammatory role, TJ-41 may improve liver autophagy and maintain energy homeostasis [46], suggesting its potential as a supportive therapy for malnutrition, sarcopenia, and cachexia. Building on this evidence, we designed this prospective randomized controlled trial to evaluate the effects of TJ-41, including its impact on circulating mitochondrial function-related factors through serial blood sampling.

This study has several limitations. Firstly, the distinct taste and odor of the multi-herbal Kampo formula meant that blinding was not possible, necessitating an open-label design. Therefore, the potential for performance bias cannot be entirely excluded. Secondly, to ensure statistical robustness given our target sample size, we were limited to two stratification factors. Although we initially considered including baseline CRP in the randomization schema, we decided against it in order to maintain statistical balance. To mitigate potential residual confounding factors, baseline CRP will be included as a covariate in the final statistical analysis.

In conclusion, this study provides an integrated approach using Kampo medicine, enabling the simultaneous clarification of its clinical significance and biological significance.

## Data Availability

The datasets generated and analyzed during the AMATERAS study are not publicly accessible, but they are available upon reasonable request to the corresponding author.
